# Which groups affected by Potentially Traumatic Events (PTEs) are most at risk for a lack of social support? A prospective population-based study on the 12-month prevalence of PTEs and risk factors for a lack of post-event social support

**DOI:** 10.1371/journal.pone.0232477

**Published:** 2020-05-29

**Authors:** Peter G. van der Velden, Ivan Komproe, Carlo Contino, Marika de Bruijne, Rolf J. Kleber, Marcel Das, Henk Schut

**Affiliations:** 1 CentERdata, Tilburg, The Netherlands; 2 Tilburg University’s Network on Health and Behavior (Nethlab), Tilburg, The Netherlands; 3 HealthnetTPO, Amsterdam, The Netherlands; 4 Faculty of Social and Behavioral Sciences, Utrecht University, Utrecht, The Netherlands; 5 Victim Support Foundation (FSH), Den Haag, The Netherlands; 6 Clinical Psychology, Faculty of Social and Behavioral Sciences, Utrecht University, Utrecht, The Netherlands; 7 Foundation Arq, Diemen, The Netherlands; Monash University, AUSTRALIA

## Abstract

**Objectives:**

Little is known about the 12-month prevalence of potentially traumatic events (PTEs) and to what extent the type of PTE is a risk factor for post-event lack of social support. In addition, it is largely unknown if pre-event mental health problems and loneliness, and demographics are risk factors for a lack of support. Aim of the present prospective study is to fill these gaps in evidence-based knowledge.

**Methods:**

A survey was conducted among a large random sample of the Dutch adult population (i.e. the longitudinal LISS panel) in March-April 2018, and linked with pre-event mental health and loneliness data from surveys conducted in 2016 (n = 5,879). We distinguished four forms of perceived social support: emotional and esteem support, and social recognition and general disapproval.

**Results:**

Loss of a significant other and/or colleague (28%) was the most prevalent 12-month PTE. The 12-month prevalence of violence, accidents and/or, and theft-related events was 13%. Multivariate logistic regression analyses revealed no differences in lack of emotional and esteem support, or in lack of recognition across non-death PTEs and death-related PTEs. However, victims of threat and physical (sexual) violence more often faced disapproval than those affected by burglary and accidents. Results furthermore showed that pre-event mental health problems, pre-event loneliness and stress during the PTE were important independent predictors of forms of support and acknowledgment. Affected individuals with a non-Western background more often lacked support and acknowledgment.

**Conclusions:**

Many adults are confronted with a PTE during a year. In general, pre-event factors and stress during the event are better predictors of a perceived lack of support and acknowledgment than type of event. Early screening programs should especially assess pre-event mental health and loneliness, besides levels of stress during the event, to identify affected people who are at risk for a lack of social support and acknowledgment.

## Introduction

Most people are confronted with potentially traumatic events (PTEs) in their lives, such as accidents, (sexual) violence and sudden death of a significant other. The effects of PTEs on mental health, especially posttraumatic stress disorder (PTSD), are well-documented, indicating that in general a minority will develop a mental disorder. Research has also shown that the prevalence of PTSD or PTSD-symptom levels vary considerably between PTEs with relatively high prevalence rates for sexual abuse and violence and relatively low rates for man-made disasters [1–4].

Perceived social support may play, as it fits the temporary and varying needs of the affected individuals, an important protective role against the development of mental health problems such as PTSD and PTSD symptoms. Social support may provide a buffer for affected individuals against these problems when dealing with PTEs [5–10]. It is therefore important to understand which PTEs put affected individuals more at risk for a lack of social support and social recognition. Many trauma studies focus on social support after PTEs, such as traffic accidents and disasters [11–21]. However, due to methodological differences between studies it is difficult to determine which groups affected by PTEs run a higher risk of perceived lack of social support. A specific form of perceived support is social acknowledgment or validation of the victims’ event-related thoughts, behavior, and feelings; i.e., positive individual or societal reactions that recognize their traumatic experiences and current difficulties. It differs from social support as an interactional process in that it includes the entire perceived societal context and not only the support from a person´s direct environment. Relatively few studies have assessed event-related social acknowledgment by the social environment [4, 22–25].

To the best of our knowledge, no study has analyzed differences in support after different PTEs. Population-based studies such as the WHO Mental Health Surveys [4, 26] and epidemiological studies [2, 27, 28] were primarily aimed at the life-time prevalence of PTEs or the prevalence of PTSD and related risk-factors, but not at differences in social support after diverse PTEs. Longitudinal population-based studies, such as the large Australian Household, Income and Labour Dynamics in Australia (HILDA) panel, have examined for instance the 12-month prevalence of life-events including several PTEs, general mental health and social support, but not event-related PTSD-symptomatology or event-related social recognition [5,9,29].

Studies focusing on more or less recent PTEs, such as PTEs during the past 12 months, are more suited than life-time studies on PTEs to assess which groups affected by PTEs run a higher risk of lack of social support because of the limited time-frame. However, a literature search using PUBMED did not identify a single population-based study on more or less recent PTEs, such as during the past 12 months, assessing lack of social support, besides studies based on local populations or communities [30]. Nevertheless, understanding which groups affected by PTEs more often suffer from a perceived lack of social support and social acknowledgment is relevant to the prioritization of health care workers in the personal and social environment [10], as well as to policymakers and public leaders [31].

Furthermore, understandably, almost all studies on social support were conducted after PTEs. For this reason, it is largely unknown to what extent pre-event factors, such as existing mental health problems and existing loneliness, are associated with lack of post-event social support and event-related social recognition. Prospective research has shown that existing mental health problems and existing loneliness were associated with post-event mental health problems and loneliness [32, 33, 34]. For example, a recent study showed that existing mental health problems and loneliness independently predicted loneliness about one and two years post-event [34], suggesting that it may negatively impact post-event social support and acknowledgment. In addition, little is known about the associations between demographics on the one hand and a lack of support and event-related recognition on the other. However, given the associations between demographic factors and PTSD-symptomatology [18, 35, 36], it is reasonable to assume that one or more of these factors may also be associated with a lack of support and recognition.

The aim of the present study is to fill these gaps in evidence-based knowledge. To identify persons who were recently affected by PTEs, we first assessed the 12-month prevalence of PTEs. Research questions were:

1. What is the 12-month prevalence of PTEs in the adult population and the prevalence of high PTSD-symptom levels across different PTEs?
2. Which PTEs are associated with a higher risk of a perceived lack of social support and event-related social acknowledgment?
3. Are pre-event mental health problems and loneliness, demographics, perceived stress during the PTE and time passed since the PTE, associated with a higher risk of a perceived lack of social support and social recognition? To improve the readability, hereafter we abbreviated ‘perceived support’ and ‘perceived social acknowledgement’ into ‘support’ and ‘acknowledgement’ as much as possible.

## Materials and methods

Data were collected using the Longitudinal Internet studies for the Social Sciences panel (LISS panel), administered by CentERdata, the Netherlands [37]. The LISS panel is a central element of the *Measurement and Experimentation in the Social Sciences-* project, funded by the Netherlands Organization for Scientific Research (NWO). The panel is based on a large representative sample drawn from the Dutch population register by Statistics Netherlands (N~7,500). Respondents who do not have a computer and/or Internet access are provided with the necessary equipment. Panel members receive an incentive of 15 euros per hour for their participation. In accordance with the new General Data Protection Regulation (GDPR) participants gave explicit consent for the use of the collected data for scientific and policy relevant research. Further information about all conducted surveys and regulations for free access to the data can be found at www.lissdata.nl (in English). The LISS panel has received the international Data Seal of Approval (see https://www.datasealofapproval.org/en/).

For the present study we extracted and linked data of respondents from several surveys. We first extracted data on previous loneliness from the annual Social Integration and Leisure Core study conducted in October 2016 (with reminders in November: T1, N^total sample^ = 6,380, response = 85.7%) and on previous mental health problems from the annual Health Core Study conducted in November 2016 (with reminders in December: T2, N^total sample^ = 6,336 response = 84.7%). In March 2018 (with reminders in April: T3) almost 7,300 participants were administered a questionnaire especially developed for this study. In total 5,989 completed the questionnaire (N^total sample^ = 7,292, response = 82.1%). For this paper we used the data of participants of 18 years and older (n = 5,879).

Our study and questionnaire was approved by an Internal Review Board (IRB) of CentERdata, consisting of independent internal and external reviewers of CentERdata. These reviewers were not involved in the research program. Since our research did not impose certain (experimental) behavior our research did not need the approval of a Dutch Medical Ethical Testing committee (METC) according to the Dutch Law (see https://english.ccmo.nl-/investigators/legal-framework-for-medical-scientific-research/your-research-is-it-subject-to-the-wmo-or-not). However, is should be noted that METC approvals for research and questionnaires among adults may be required in the Netherlands when for instance the (expected) burden for all respondents is (very) large. In case of doubt, METC’s can help to examine if the proposed research project needs a formal METC approval or not. Yet, two similar trauma studies with semi-structured interviews and questionnaires were evaluated in the past by an METC (METC UMC Utrecht University, the Netherlands) as not requiring a METC approval (AvG/mvdl/03/326 and AvG/mm/06/14497). Furthermore, previous and similar research on trauma using the same LISS panel [38] demonstrated that the possible burden of participating in research on trauma is not trauma-related, i.e. not related to the level of posttraumatic stress symptoms, type of trauma, or trauma-related coping self-efficacy. Importantly, a recent meta-analysis concluded that “…findings suggest that trauma-related research can continue without harming participants” [39]. For these reasons we did not ask an METC for advice or a formal METC approval, besides the IRB approval. In accordance with the new General Data Protection Regulation (GDPR) participants gave explicit consent for the use of the collected data for scientific and policy relevant research.

Due to a refreshment sample to update the LISS panel, at T3 about 900 new respondents could be selected and included on top of the number respondents at T1/T2.

### Measures

#### Potentially traumatic events

The 12-month prevalence of PTE was examined by means of a list of 21 events (1 = yes, 2 = no) based on previous research on such events [29, 38, 40, 41]. These included events such as severe threat without physical violence, events with physical violence, accidents, but also the (un)expected death of a significant other (partner, family, friend) based on Criterion A1 events in DSM-IV and events in the ICD-11 (see Table 2 below for overview). Since we focused on adults and events in the past twelve months, PTEs such as adverse child experiences were excluded. Participants could describe PTEs in the past twelve months that were not listed. They were recoded in terms of present or new categories of PTE.

If respondents experienced one PTE in the past 12 months, respondents were asked to keep this event in mind when answering the event-related questions (such as on stress during the PTE and PTSD symptoms). This event was displayed on the screen when the event-related questions (about PTSD, disclosure, and recognition) were administered. In case respondents reported two or more events, respondents were asked to rate the most adverse or stressful one. As we were not able to recode the open answers while respondents participated, we could not randomize and select one PTE for the respondents to focus on during the event-related questions [26]. We therefore used the following strategy: when respondents had also been confronted with a serious disease, (expected or unexpected) death of a significant other or colleague and another event (such as violence, threat, or accident), respondents were automatically asked to keep the non-illness/non-death event in mind when answering the event-related questions such as stress during the event and PTSD symptoms.

Participants were asked when the (most drastic or stressful) PTE in the past 12 months took place (1 = one week ago to 8 = 7–12 months ago), and to rate the level of stress during the event (1 = not or almost not to 5 = very much). For the present study scores were recoded as very stressful (4,5) or not very stressful (1,2,3).

#### Posttraumatic stress symptoms

PTSD-symptomatology following the (most stressful or selected) PTE among affected respondents was assessed using the 8-items version of the PCL-5 [42–44] that assessed symptoms across the four symptom clusters of PTSD according to DSM-5 (APA, 2013)[45]. Items have 5-point Likert scales and focus on symptoms in the past month (1 = not at all to 5 = extremely; Cronbach’s Alpha = .92).

#### Disclosure of events

Since support and acknowledgment cannot be offered by others if they are not informed or aware of the event the participant was confronted with, we first assessed whether the participants communicated with others about the event (displayed on screen) using a list of 20 persons and organizations (varying from family to Victim Support Netherlands). If a participant indicated that he/she had not revealed the PTE, we asked if other people knew or were aware that they were affected (1 = yes, one or more persons, 2 = yes, I suppose one or more persons, 3 = no, nobody (except possible perpetrator), 4 = I don’t know). When affected participants reported “no, nobody (except possible perpetrator)” or “I don’t know”, the questionnaire about recognition was not administered.

#### Post-event lack of social support

Two scales of the Social Support List—Discrepancy (SSL–D) [46, 47] were administered at T3 to all respondents to assess a perceived lack of emotional support in response to problems (8 items) and lack of esteem support (6 items). The SSL-D invites respondents to rate their opinions or perceptions about people with whom they interact on 4-point scales (1 = I miss it, I would like it to happen more often to 4 = it happens too often). Low scores indicate a greater lack of support (Cronbach’s Alphas ≥ .78).

#### Lack of post-event social acknowledgment

The Social Acknowledgment Questionnaire (SAQ) [8, 48] was administered to examine event-related general disapproval and social recognition. The SAQ has positively and negatively formulated items with 5-point answer categories (1 = totally disagree to 5 = totally agree). We used the 5-item general disapproval scale (Cronbach’s Alpha = 0.86) that consists of items such as “There is not enough sympathy for what happened to me” and “Most people cannot understand what I went through”. We furthermore used the 6-item recognition scale (Cronbach’s Alpha = 0.77) that consists of items such as “My friends feel sympathy for what happened to me” and “The reactions of my acquaintances were helpful”. This scale also included an item on important public figures in the respondent’s place of residence (e.g. mayor, priest) and superiors, but for the present study these two items were omitted because these figures might not have known/been aware be of the PTEs that the respondent experienced.

#### Pre-event loneliness

Loneliness was assessed at T1 using the six-item of the Loneliness Scale [49, 50]. Respondents were asked to rate items on three-point Likert scales (1 = yes to 3 = no). We calculated the total score after recoding the three negatively formulated items (Cronbach’s Alpha’s = 0.83). Loneliness scores were recoded into very low (18), low (17), medium (15,16) and high (6–14) levels of loneliness to obtain 4 more or less equal sized subgroups with different levels of loneliness. Respondents who did not participate at T1 were coded as “unknown” (see Tables 3 and 4).

#### Pre-event mental health problems

Anxiety and depressive symptoms (hereafter labeled Mental Health Problems, MHP) were examined at T2 using the 5-item Mental Health Index or Inventory (MHI-5) [51, 52]. Respondents were asked to rate their mental health during the past month on questions with a 6-point response format (1 = never to 6 = continuously). After recoding the third and fifth item, the total scores were computed (Cronbach’s Alpha = .86). For the current study, scores were recoded, based on quartiles, into very low (0–8), low (9–10), medium (11–14) and high levels of MHP (15 and higher). Respondents who did not participate at T2 were again coded as “unknown” (see Tables 3 and 4).

#### Data analysis

We first assessed 32 exclusive demographic profiles among the total adult Dutch population (N^2016^ = 13,734,958), based on data from Statistics Netherlands. We used Statistics Netherlands data of April 2016 because 2016 offered the most recent data on all demographic characteristics and this study was conducted in March-April. The 32 profiles were constructed using the following demographic characteristics: gender (2 categories), age categories (4 categories), marital status (2 categories), and employment status (2 categories) totaling 2*4*2*2 = 32 exclusive demographic profiles. All results are based on the weighted sample.

To answer research question 1, the prevalence of a specific PTE was computed by dividing the number of respondents who reported a specific PTE by the total number of participants. We computed the prevalence of the selected/most adverse PTE and 95% confidence intervals of this prevalence and the prevalence of high levels of PTSD-symptoms for each selected/most adverse PTE. Differences in prevalence of high levels of PTSD-symptoms between types of events were assessed using multivariate logistic regression analyses, while controlling for age categories, gender, employment status, marital status, education, background, loneliness at T1, mental health at T2, and time since event.

Research questions 2 and 3 were answered simultaneously using multivariate logistic regression analyses: type of event, and all other predictors were entered simultaneously. Multivariate logistic regression analyses (MLRA) were used instead of multiple regression analyses because several study variables did not present linear scales, such as type of event (selected/most adverse PTE), period in which event took place, ethnical background and education. In addition, MLRA enabled us to code and include respondents who did not participate at T1 or T2 (the refreshment sample). Dependent variables were lack of social support (emotional and esteem support), and acknowledgment (social recognition and general disapproval) at T3. For the present study, scores on the dependent variables (i.e. the scales of the SSL-D and SAQ) were therefore dichotomized in high and low scores (cut-off scores of about lower 20% or lowest two deciles among the total study sample: SSL-D^high lack of emotional support^ scores ≤ 19, SSL-D^high lack of esteem support^ scores ≤ 14; upper two deciles SAQ^high lack social recognition^ scores ≥ 15; lower two deciles SAQ^high general disapproval^ scores ≤ 11). The PCL8-scores were also dichotomized based on cut-off of about the 80^th^ percentile into low and high PTSD-symptom levels. We used this cut-off because to date no Dutch clinically validated cut-off is available for the PCL 8-item version.In sum, predictors in the multivariate logistic regression analyses were: type of event, time of event, stress during event, pre-event mental health problems and loneliness, and demographics.

All logistic regression analyses were conducted separately among those affected by violence (sexual, physical or threat), accidents (traffic or medical) and (online) theft-related events (VAT-PTE group) and those affected by death-related events (DR-PTE group). The physical disease subgroup, mainly consisting of respondents with serious diseases like cancer, did not fit in either the VAT-PTE or DR-PTE subgroup and were therefore omitted from the analyses.

## Results

The characteristics of the Dutch adult population and respondents before and after weighting are presented in Table 1.

**Table 1:** Characteristics general population and respondents before and after weighting.

	18 years or older				
	Adult Dutch population^1^	Participants		Participants weighted^2^	
	(N = 13,734,958)	(N = 5,879)		(N = 5,879)	
	%	N	%	N	%
Gender					
• man	49.2	2716	46.2	2893	49.2
• woman	50.8	3163	53.8	2986	50.8
Age (years) T3					
• 18–34	25.9	1227	20.9	1525	25.9
• 35–49	24.5	1302	22.1	1442	24.5
• 50–64	25.9	1631	27.7	1521	25.9
• 65 or older	23.7	1719	29.2	1392	23.7
Marital status T3					
• not married	52.7	2834	48.2	3096	52.7
• married	47.3	3045	51.8	2783	47.3
Employment status T3					
• not employed	47.0	2946	50.1	2765	47.0
• employed	53.0	2933	49.9	3114	53.0
Highest education level T3					
• primary educ^3^.	n.a.	225	3.8	215	3.7
• preparatory intermediate vocational educ.	n.a.	1113	18.9	1020	17.3
• higher general secondary/pre-university educ.	n.a.	393	6.7	381	6.5
• intermediate professional educ.	n.a.	1363	23.2	1376	23.4
• higher professional educ.	n.a.	1717	29.2	1734	29.5
• university	n.a.	1068	18.2	1153	19.6

^1^ Based on data from Statistics Netherlands of Dutch population, April 2016.

^2^ The sample was weighted on the distribution of 32 profiles of the total adult Dutch population, based on gender (2 categories), age (4 categories), marital status (2 categories), and employment status (2 categories) totaling 2*4*2*2 = 32 exclusive profiles.

^3^ Including other education and no education (yet). n.a. = not available for total adult Dutch population. T3 = March-April 2018. educ. = education.

### 12-month prevalence of PTE and PTSD-symptomatology

The 12-month prevalence rates of PTEs are shown in Table 2. The events are arranged into 11 main categories (in bold) with varying numbers of sub categories. The table shows that about 40% of the respondents was confronted with one or more PTE in the past 12 months (and 7.0% with other life-events). The 12-month prevalence of physical violence, not by own partner was 1.17%, and the 12-month prevalence of any physical (sexual) violence event 2.01%. The 12-month prevalence of any violence, accident, theft, and threat-related event was 13.4%. The expected death of a significant other was the most prevalent PTE (15.4%). The prevalence of any death-related event (significant other, colleague and/or dying) was 27.3%. The numbers of “any” are lower than the total number of forms of a specific type of PTE, indicating that some respondents were confronted with more than one form of PTE (of the specific type of PTE).

**Table 2:** 12-Month prevalence of potentially traumatic events.

		PTE in past 12 months		
	Confronted with		Single/most adverse event	
	n^total^	%^total^ (95% CI)	n^total^	%^total^ (95% CI)
No PTE in past 12 months	3,495	59.45 (58.19–60.70)		n.a.
**Severe threat (any)**	**199**	**3.38 (2.95–3.88)**	**140**	**2.38 (2.02–2.80)**
• severe threat without physical violence	151	2.57 (2.19–3.00)	104	1.77 (1.46–2.14)
• online threat	68	1.16 (0.91–1.46)	35	0.60 (0.43–0.83)
• verbal aggression^1^	1	0.02 (0.00–0.10)	0	0.00 (0.00–0.07)
• sexual intimidation^1^	1	0.02 (0.00–0.10)	1	0.02 (0.00–0.10)
**Traffic accident**	**185**	**3.15 (2.73–3.62)**	**150**	**2.55 (2.18–2.98)**
**Other accidents/disasters (any)**	**88**	**1.50 (1.22–1.84)**	**56**	**0.95 (0.73–1.23)**
• airplane accident	7	0.12 (0.06–0.25)	1	0.02 (0.00–0.10)
• accident at work	46	0.78 (0.59–1.04)	27	0.46 (0.32–0.67)
• fire	42	0.71 (0.53–0.96)	27	0.46 (0.32–0.67)
• natural disaster	2	0.03 (0.01–0.12)	1	0.02 (0.00–0.10)
**Medical accident/error**	**105**	**1.79 (1.47–2.15)**	**84**	**1.43 (1.15–1.76)**
**Physical (sexual) violence (any)**	**118**	**2.01 (2.21–3.02)**	**84**	**1.43 (1.15–1.76)**
• sexual violence or abuse	17	0.29 (0.18–0.46)	12	0.20 (0.12–0.36)
• online sexual violence or abuse	18	0.31 (0.19–0.48)	8	0.14 (0.07–0.27)
• robbery	16	0.27 (0.17–0.44)	7	0.12 (0.06–0.25)
• physical violence, not by own partner	69	1.17 (0.93–1.48)	41	0.70 (0.51–0.94)
• physical violence, by own partner	28	0.48 (0.33–0.69)	14	0.24 (0.14–0.40)
• various physical violence^1^	4	0.07 (0.03–0.17)	2	0.03 (0.01–0.12)
**Burglary, fraud and theft (any)**	**191**	**3.25 (2.83–3.73)**	**148**	**2.52 (2.14–2.94)**
• burglary	82	1.39 (1.12–1.72)	55	0.94 (0.72–1.21)
• theft or fraud	128	2.18 (1.83–2.58)	93	1.58 (1.29–1.93)
**Online theft and fraud**	**141**	**2.40 (2.03–2.81)**	**107**	**1.82 (1.50–2.19)**
**Death significant other (any)**	**1,370**	**23.30 (22.24–24.40)**	**1,091**	**18.56 (17.5–19.48)**
• death significant other, expected	907	15.43 (14.46–16.30)	583	9.92 (9.13–10.66)
• death of significant other, unexpected	716	12.18 (11.31–12.98)	502	8.54 (7.81–9.24)
• dying or death significant other^1^	18	0.31 (0.19–0.48)	6	0.10 (0.05–0.22)
**Death colleague (any)**	**338**	**5.75 (5.18–6.37)**	**182**	**3.10 (2.67–3.55)**
• death colleague, expected	165	2.81 (2.40–3.24)	63	1.07 (0.83–1.36)
• death colleague, unexpected	224	3.81 (3.33–4.31)	119	2.02 (1.68–2.40)
**Physical disease (any)**	**220**	**3.74 (3.29–4.26)**	**124**	**2.11 (1.77–2.50)**
• serious disease (cancer, etc.)	208	3.54 (3.08–4.03)	120	2.04 (1.70–2.43)
• serious infection (HIV, aids, etc.)	10	0.17 (0.09–0.31)	0	0.00 (0.00–0.07)
• became very ill^1^	8	0.14 (0.07–0.27)	4	0.07 (0.03–0.17)
**Various other life events**	**410**	**6.97 (6.31–7.61)**	**220**	**3.74 (3.27–4.23)**

^1^ These events were added after recoding the open answers. 95% CI = 95% confidence interval of prevalence. Very stressful = event(s) in category rated as much or very much stressful during event. n.a = not applicable. Categories of PTE’s are presented in **bold.** PTE = potentially traumatic events.

The prevalence of high PTSD-symptom levels among the distinguished subgroups within the VAT-PTE and DR-PTE group are presented in Table 3. To prevent lengthy tables with empty rows as much as possible, the results with respect to the VAT-PTE subgroups are presented in the same row as the results of the DR-PTE subgroups (but in different column). We used the “//” characters to separate the subgroups in the row of the first column. This format was also applied in Tables 4 and 5.

**Table 3:** PTSD-symptomatology among affected groups.

	High PTSD-symptom levels			
	affected by violence, accidents and theft related events (N = 767)		affected by death related events (N = 1,266)	
	%	AOR (95% CI)	%	AOR (95% CI)
**VAT-PTE// DR-PTE**				
• burglary, theft and fraud// death coll. exp. (ref.)	12.5***	1	7.8***	1
• accidents// death coll. unexp.	15.0	1.11 (0.59–2.08)	5.9	0.48 (0.13–1.76)
• medical error// death sign. other exp.	34.5	2.37 (1.13–4.96)*	13.5	1.27 (0.43–3.69)
• (online) threat// death sign. other unexp.	33.6	3.99 (2.15–7.39)***	18.1	1.52 (0.52–4.46)
• physical (sexual) violence	47.6	5.50 (2.78–10.86)***		

VAT-PTE = violence (sexual, physical or threat), accidents (traffic or medical) and (online) theft-related event. DR-PTE = death-related events. Coll. exp. = colleague expected. Coll. unexp. = colleague unexpected. Sign. other exp. = significant other expected. Sign. other unexp. = significant other unexpected. Ref. = reference group. AOR = Odds ratio adjusted for age, gender, employment status, marital status, education, background, loneliness at T1, mental health at T2, and stress during and time since event. Results are based on the weighted sample.

* p < 0.05,

** p <0.01,

*** p <0.001

**Table 4:** Predictors of lack of emotional and esteem support.

	Lack of emotional support T3				Lack of esteem support T3			
	affected by violence, accidents, and theft related events		affected by death related events		affected by violence, accidents, and theft related events		affected by death related events	
	(n = 767)		(n = 1,266)		(n = 767)		(n = 1,266)	
	%	AOR	%	AOR	%	AOR	%	AOR
**Age categories (in years)**								
• 18–34 (ref.)	28.3	1	15.5	1	26.6	1	15.5	1
• 35–49	32.7	1.38	21.2	1.48	29.6	1.04	18.6	1.12
• 50–64	26.2	1.24	19.8	1.54	22.3	0.88	16.5	1.09
• 65 and older	28.2	1.52	18.5	1.46	23.4	0.90	12.9	0.85
**Gender**								
• man (ref.)	27.9	1	17.5	1	27.9	1	16.9	1
• woman	30.2	0.83	19.9	0.95	23.0	0.52***	14.5	0.78
**Employed**								
• no (ref.)	33.1*	1	20.4	1	30.4**	1	15.5	1
• yes	25.8	0.92	17.0	1.01	21.9	0.68	15.8	1.04
**Married**								
• no (ref.)	31.2	1	20.6	1	25.8	1	16.6	1
• yes	25.3	1.01	16.9	0.91	25.3	1.70*	14.7	1.03
**Highest education level**								
• prim. educ. (ref.)	33.1	1	24.7***	1	25.8	1	20.1	1
• higher gen. sec.	34.7	1.57	29.7	1.33	30.6	1.49	12.1	0.46*
• inter. prof. educ.	33.5	1.25	17.9	0.70	29.2	1.28	16.0	0.65
• higher prof. educ.	26.2	1.08	14.3	0.56**	23.4	1.15	13.6	0.55
• university	22.8	0.86	16.2	0.67	23.5	1.10	14.9	0.60
**Background**								
• Dutch origin (ref.)	24.7***	1	18.1***	1	22.1	1	14.1**	1
• West-European	33.0	1.40	17.8	0.89	33.0	2.04**	18.6	1.39
• Non-Western	44.3	1.81*	24.7	1.24	35.1	1.43	29.5	2.36**
• unknown	37.5	1.61	27.0	1.96	30.0	1.15	16.7	1.27
**Loneliness T1**								
• very low (ref.)	11.2***	1	11.2***	1	10.7***	1	9.3***	1
• low	13.2	0.92	16.4	1.36	8.8	0.56	11.8	1.16
• medium	28.6	1.92	21.8	1.58	26.3	1.79	18.5	1.74*
• high	44.4	2.80**	39.7	2.72***	34.7	1.81	30.7	2.65***
• unknown^1^	38.0	2.79*	19.5	1.77	35.8	2.26	18.0	1.90
**Mental health problems T2**								
• not (ref.)	6.5***	1	6.6***	1	2.8***	1	7.7***	1
• low	12.1	1.92	15.0	2.17*	9.9	4.36*	12.0	1.44
• medium	24.8	4.53***	22.3	3.40***	23.0	12.57***	16.0	1.66
• high	43.8	7.25***	36.7	5.68***	36.3	18.47***	28.9	3.11***
• unknown^1^	37.7	4.18**	19.1	2.44*	35.9	12.64***	17.2	1.43
**Period event**								
• up to 1 months ago (ref.)	33.3	1	21.8	1	27.9	1	18.4	1
• 1–2 months ago	29.8	0.81	17.5	0.65	26.6	0.81	15.6	0.69
• 3–4 months ago	23.0	0.56*	16.0	0.71	18.9	0.52*	12.1	0.60
• 5–6 months ago	26.2	0.59	16.5	0.71	32.7	1.06	17.6	0.89
• 7–12 months ago	30.5	0.72	19.2	0.73	24.2	0.61	14.4	0.65*
**Very stressful during event**								
• no (ref.)	23.1***	1	16.3***	1	20.4***	1	15.4	1
• yes	39.4	1.96**	25.4	1.49*	34.9	1.64*	16.6	0.98
**VAT PTE// DR-PTE**								
• burglary, etc. // death coll. exp. (ref.)	24.2*	1	14.3	1	23.8**	1	12.7	1
• accidents// death coll. unexp.	26.2	1.17	18.5	1.08	22.9	1.01	15.3	1.11
• medical error// death sign. other exp.	33.3	0.99	17.0	1.02	34.5	1.22	16.5	1.42
• (online) threat// death sign. other unexp.	32.1	1.31	21.5	1.39	20.1	0.74	15.1	1.23
• physical (sexual) violence	40.5	1.58			38.1	1.55		

VAT-PTE = violence (sexual, physical or threat), accidents (traffic or medical) and (online) theft-related event. DR-PTE = death-related events.

^1^ Unknown: information not available because they were new participants that were recruited after T1/T2 or did not participate at T1/T2 for another reason. At T3 about 900 more respondents could be selected than at T1/T2. ref. = reference group. AOR = Odds ratio adjusted for all predictors in Table 4. % = percentage of respondents with relative high scores on lack of corresponding support. prim. educ./prep. inter. voc. educ = primary education or preparatory intermediate vocational education; higher gen. sec. pre-uni educ. = higher general secondary/pre-university education; inter. prof. educ. = intermediate professional education; higher prof. educ. = higher professional education. Results are based on the weighted sample.

* p <0.05,

** p <0.01,

*** p <0.001.

**Table 5:** Predictors of general disapproval and lack of social recognition.

	General disapproval T3				Lack of social recognition T3			
	affected by violence, accidents, and theft related events		affected by death related events		affected by violence, accidents, and theft related events		affected by death related events	
	(N = 735)		(n = 1,245)		(n = 735)		(n = 1,245)	
	%	AOR	%	AOR	%	AOR	%	AOR
**Age categories (in years)**								
• 18–34 (ref.)	19.3	1	14.2	1	23.1	1	16.5	1
• 35–49	26.6	1.86*	16.3	0.95	20.7	0.94	17.2	0.86
• 50–64	24.0	1.80*	15.7	1.02	29.7	1.54	17.8	0.84
• 65 and older	22.2	1.40	15.7	1.21	26.3	1.35	19.8	1.03
**Gender**								
• man (ref.)	21.1	1	14.8	1	28.1*	1	21.6**	1
• woman	24.9	0.97	16.2	0.90	20.8	0.59**	14.9	0.74
**Employed**								
• no (ref.)	28.1**	1	16.5	1	26.2	1	18.2	1
• yes	18.6	0.88	14.5	1.19	23.4	0.86	17.9	0.95
**Married**								
• no (ref.)	25.9**	1	16.7	1	24.2	1	15.0**	1
• yes	17.6	0.70	14.2	0.89	25.4	1.17	21.4	1.57**
**Highest education level**								
• prim. educ. (ref.)	30.8*	1	20.4**	1	22.5	1	17.5	1
• higher gen. sec.	19.6	0.52	19.3	0.91	26.1	1.72	13.6	0.71
• inter. prof. educ.	26.4	0.93	19.1	0.97	23.7	1.27	20.5	1.12
• higher prof. educ.	17.7	0.77	12.1	0.55*	25.5	1.59	18.6	1.03
• university	20.6	0.87	10.4	0.44**	25.0	1.34	16.7	0.89
**Background**								
• Dutch origin (ref.)	20.0**	1	14.5	1	23.6	1	19.0	1
• West-European	20.6	0.71	17.2	1.23	28.1	1.38	16.4	0.91
• Non-Western	36.8	1.95*	25.6	1.94*	25.3	1.30	12.6	0.65
• unknown	31.6	2.28	13.5	0.93	28.2	1.65	11.1	0.68
**Loneliness T1**								
• very low (ref.)	8.9***	1	9.9***	1	17.8***	1	14.5*	1
• low	11.1	1.01	15.3	1.58	14.3	0.63	18.0	1.28
• medium	23.5	2.37*	17.4	1.55	31.3	1.68	22.3	1.73*
• high	39.5	3.36**	29.3	2.26**	33.6	1.88	24.8	2.20**
• unknown^1^	27.1	3.04*	16.5	2.31	24.8	2.48	17.4	1.75
**Mental health problems T2**								
• not (ref.)	10.6***	1	9.7***	1	16.3*	1	17.8	1
• low	13.8	1.21	13.0	1.24	17.0	1.13	18.6	1.05
• medium	17.6	1.45	15.3	1.36	30.1	2.46***	17.1	0.89
• high	37.7	2.39*	28.7	2.71**	31.9	2.32*	21.7	1.25
• unknown^1^	25.9	1.13	15.3	0.88	23.6	0.99	16.9	0.84
**Period event**								
• up to 1 months ago (ref.)	21.2	1	14.6	1	25.2	1	22.4	1
• 1–2 months ago	19.3	0.85	17.8	1.19	14.8	0.51	15.1	0.61
• 3–4 months ago	16.8	0.84	17.1	1.31	24.5	0.99	15.6	0.71
• 5–6 months ago	21.4	1.00	13.1	0.89	28.2	1.28	17.2	0.76
• 7–12 months ago	29.0	1.39	15.5	0.96	26.1	1.23	18.5	0.98
**Very stressful during event**								
• no (ref.)	15.5***	1	12.7***	1	26.5	1	21.2***	1
• yes	36.1	1.77***	22.9	1.84***	21.0	0.65*	10.0	0.44***
**VAT PTE// DR-PTE**								
• burglary, etc.// death coll. exp. (ref.)	13.0***	1	11.3	1	26.8	1	27.4*	1
• accidents// death coll. unexp.	13.4	1.04	16.2	1.29	20.3	0.73	27.0	0.99
• medical error// death sign. other exp.	40.2	3.51***	14.2	1.11	18.3	0.63	17.0	0.59
(online) threat// death sign. other unexp.	31.0	3.19***	17.5	1.30	26.4	1.03	16.3	0.62
• physical (sexual) violence	46.7	5.32***	11.3		33.3	1.66		

VAT-PTE = violence (sexual, physical or threat), accidents (traffic or medical) and (online) theft-related event. DR-PTE = death-related events.

^1^ Unknown: information not available because they were new participants that were recruited after T1/T2 or did not participate at T1/T2 for another reason. At T3 about 900 more respondents could be selected than at T1/T2. ref. = reference group. AOR = Odds ratio adjusted for all predictors in Table 5. % = percentage of respondents with relative high scores on lack of corresponding support. prim. educ./prep. inter. voc. educ = primary education or preparatory intermediate vocational education; higher gen. sec. pre-uni educ. = higher general secondary/pre-university education; inter. prof. educ. = intermediate professional education; higher prof. educ. = higher professional education. Results are based on the weighted sample.

* p <0.05,

** p <0.01,

*** p <0.001.

Table 3 shows that among the VAT-PTE group the prevalence of high PTSD-symptom levels was 12.5% for victims of burglary, theft and fraud, 15.0% for accidents, 34.5% for medical mistakes/errors, 33.6% for (online) threat, and 47.6% for physical (sexual) violence (see Table 3). The last three subgroups had significantly more often high levels of PTSD symptoms than victims of burglary, theft, and fraud, when controlling for demographics, pre-event functioning and time since event. Among the DR-PTE group the prevalence of PTSD symptoms was 7.8% for respondents confronted with an expected death of a colleague, 5.9% for unexpected death of a colleague, 13.5% for unexpected death of a significant other, and 18.1% for the expected death of a significant other. However, no significant differences between these groups remained when controlling for the demographics, pre-event functioning and time since event.

### Predictors of lack of emotional and esteem support

Table 4 shows that among the VAT-PTE and DR-PTE group, no type of event was independently significantly associated with a higher risk of lack of emotional or esteem support (see S1 Appendix for 95% confidence intervals and p-values). However, existing loneliness (T1) and especially mental health (T2) about 1.5 years earlier were independent predictors of current lack of social support among the VAT-PTE group and DR-PTE group. Affected respondents who experienced the PTE in the past 12 months as very stressful compared to those who did not, were more likely to experience a lack of emotional and esteem support at T3.

### Predictors of lack of social recognition and general disapproval

Table 5 shows that victims of medical accidents/errors, (online) threat and physical (sexual) violence more often reported general disapproval than victims of burglary, theft and fraud while controlling for all other variables in Table 4, but not social recognition. Among the DR-PTE group, type of event was not an independent predictor of social recognition and general disapproval (see S2 Appendix for 95% confidence intervals and p-values).

Table 5 furthermore shows that affected respondents in both the VAT and DR group with a Non-Western background more often reported general disapproval than affected Dutch natives, but that they did not differ with respect to social recognition. High levels of previous loneliness and high levels of previous mental health problems were independent predictors of general disapproval among both VAT-PTE and DR-PTE subgroups, while previous mental health predicted lack of recognition in the VAR-PTE groups and previous loneliness predicted lack of recognition in DR-PTE group. High level of stress during the event was significantly associated with general disapproval among both the VAT and DR group.

## Discussion

About 40% of the respondents in this study were confronted with one of more PTEs in the past 12 months, and in general about 30% of the investigated PTEs were experienced as very stressful. The (unexpected) death of a significant other or colleague was the most prevalent 12-month PTE (28%), which is in line with the large population-based study by Hentschel et al. (2016) where 21.5% of the respondents (assessed in 2005) were confronted with the death of a significant other (death of spouse or child, close relative/family member/friend) in the past year. However, in the Detroit Neighborhood Health Study the 12-months prevalence of the sudden, unexpected death of a close friend or relative was much higher (38.1%) [53]. These results indicate that PTEs are highly prevalent in the general population. In line with previous research, victims of severe threat and physical (sexual) violence more often had high PTSD-symptom levels than victims of burglary, theft and fraud when controlling for all other study variables [4,26]. Among the DR-PTE group, the prevalence of high PTSD symptoms levels differed significantly, but not after controlling for the same study variables.

With respect to the question which of the PTE subgroups were more at risk for a perceived lack of support and recognition, compared to other PTE subgroups, the findings revealed the following patterns. Specific types of PTEs, i.e. medical error, severe threat and physical (sexual) violence, did put victims more at risk for general disapproval when controlling for all other study variables. However, when controlling for all other study variables including event-related factors the type of PTE did not provide insight into which specific VAT-PTE and DR-PTE subgroups were more at risk for lack emotional support, lack of esteem support and social recognition, in contrast to stress during the events. Being employed was not significantly independently associated with forms of social support and social acknowledgment, while men compared to women more often reported a lack of esteem support and general recognition. With respect to education and marital status, results did not show a clear pattern in significant associations.

High levels of stress during the PTE were independent predictors of emotional support, esteem support, social recognition, and general disapproval among both the VAT-PTE and DR-PTE subgroup (except esteem support among DR-PTE subgroup). Given the disclosure rate of about 95%, we may expect that many people in the social environment were indeed informed about what happened and therefore could have provided support. We therefore may expect (or hope) that a higher stress level during the event is associated with a lower prevalence of a lack of support and recognition. Yet findings showed the opposite. A possible explanation for this finding is that victims with higher stress levels during the event more often suffer from PTSD-symptoms. Research has shown that PTSD-symptom levels erode social support and social acknowledgment, also called “social selection” [6, 54]. In an earlier study we found that potentially traumatic events have, depending on PTSD-symptom levels, negative and positive effects on post-event loneliness in favor of affected adults with very low PTSD symptoms levels [34]. People in the social environment may find it difficult to respond because of the PTE and stress reactions, and therefore avoid talking about the experiences and problems, resulting in lower support levels. Another explanation is that in situations with (sudden) higher levels of experienced stress, there is more need for support to deal with the (increased) stress than in normal situations [55, 56]. The amount of provided post-event support may be similar to the amount of pre-event support, but due to what happened to need for support increased resulting in higher lack of support levels.

Although future research is needed to substantiate these findings, the findings of this study clearly suggest that level of stress during the event is a much stronger predictor for lack of support and recognition after PTE than type of PTE. Time since the PTEs was not clearly associated with lack of support and recognition. In addition, results showed that the significant relationships between support and predictors such as pre-event mental health, loneliness and age are not linear in nature.

Besides replication of our study findings, future research should focus on the role of pre-event lack of social support in post-event lack of social support. It is unknown to what extent post-event lack of support represents pre-event lack of support after different PTE’s: prospective population-based studies addressing this important question are, to the best of our knowledge, absent. Our finding that pre-event loneliness independently predicted of lack of support and social acknowledgement may serve as an indication of a significant relationship. In addition, future prospective research with pre-event social support assessments should examine if and how changed needs affect perceived support levels across different PTEs.

### Strengths and limitations

To the best of our knowledge, this is the first population-based study focusing on 12-month prevalence of PTEs, social support (emotional and esteem) and event-related social acknowledgment (general disapproval and social recognition) encountered by victims across different PTEs, as well as focusing on predictors of support and acknowledgment. To date, very few studies have examined predictors of social support other than post-event mental health problems such as PTSD-symptoms.

The use of a large representative longitudinal population-based sample with non-retrospective pre-event measures on mental health problems and loneliness is a major strength of our present study. Our study relied less on recalled memories of PTEs compared to studies on life-time PTEs which are more sensitive to recall-bias [57, 58]. However, we did not conduct clinical interviews to examine PTSD, nor were clinical interviews conducted in the past. Leaving the problem of non-overlap between PTSD according to DSM-IV and DSM-5 aside [59, 60], such data would have enriched our study. We have no information about examined PTEs in the past years. The reports about support and acknowledgment are about perceived support and acknowledgment: we have no data about provided support and acknowledgment as perceived by the providers. Although this study is based on a large sample, a much larger sample would have enabled us to further assess support and acknowledgment among smaller subgroups such as victims of sexual violence or abuse. Since social support and PTSD symptoms were assessed at the same time (T3), it was beyond the scope of the present study to examine the interplay between social support and PTSD symptoms [61, 62].

## Conclusions

In contrast to the type of event, already existing loneliness and already existing mental health problems systematically and independently predicted a lack of emotional support, esteem support, social recognition and social disapproval among the VAT-PTE and DR-PTE subgroup, indicating that a lack of support and recognition after PTEs depends on pre-existing personal factors unrelated to the PTEs in the past 12 months. The findings clearly indicate that a lack of support and recognition are direct consequences of an existing lack of social contacts, social network or social interactions (loneliness). In addition, our findings are in line with previous research showing that pre-event mental health problems and loneliness are important independent predictors of post-event loneliness [34]. These results indicate that early screening for risk of psycho-social problems after PTEs should include an assessment of pre-event functioning to enable tailor-made interventions. Either way, results suggest that affected people who need more support and acknowledgment because of existing mental health problem and loneliness, stress levels during the event and current PTSD-symptom levels, in fact received it less, which underlines the need for interventions to improve social support and social acknowledgment.

## Supporting information

S1 Appendix Predictors of lack of emotional and esteem support. (DOCX)

S2 Appendix Predictors of general disapproval and lack of social recognition. (DOCX)
