# Supplementary material for: Which groups affected by Potentially Traumatic Events (PTEs) are most at risk for a lack of social support? A prospective population-based study on the 12-month prevalence of PTEs and risk factors for a lack of post-event social support
Source: PLoS One. 2020 May 29;15(5):e0232477. doi: 10.1371/journal.pone.0232477 (PMC7259781; doi:10.1371/journal.pone.0232477)
Supplement: S1 Appendix — (DOCX) [file pone.0232477.s001.docx]

S1 Appendix Predictors of lack of emotional and esteem support
